# Bandgap Calculation and Experimental Analysis of Piezoelectric Phononic Crystals Based on Partial Differential Equations

**DOI:** 10.3390/ma17153780

**Published:** 2024-08-01

**Authors:** Chunsheng Song, Yurun Han, Youliang Jiang, Muyan Xie, Yang Jiang, Kangchao Tang

**Affiliations:** 1 School of Mechanical and Electrical Engineering, Wuhan University of Technology, Wuhan 430070, China; song_chsh@163.com (C.S.); hanyurun_23@163.com (Y.H.); muyanxie@whut.edu.cn (M.X.); yang18674003556@163.com (Y.J.); tangkc0009@163.com (K.T.); 2 Institute of Advanced Material Manufacturing Equipment and Technology, Wuhan University of Technology, Wuhan 430070, China

**Keywords:** piezoelectric phononic crystal, PDE (partial differential equation), complex band structure, localized resonance bandgap

## Abstract

Focusing on the bending wave characteristic of plate–shell structures, this paper derives the complex band curve of piezoelectric phononic crystal based on the equilibrium differential equation in the plane stress state using COMSOL PDE 6.2. To ascertain the computational model’s accuracy, the computed complex band curve is then cross-validated against real band curves obtained through coupling simulations. Utilizing this model, this paper investigates the impact of structural and electrical parameters on the bandgap range and the attenuation coefficient in the bandgap. Results indicate that the larger surface areas of the piezoelectric sheet correspond to lower center bands in the bandgap, while increased thickness widens the attenuation coefficient range with increased peak values. Furthermore, the influence of inductance on the bandgap conforms to the variation law of the electrical LC resonance frequency, and increased resistance widens the attenuation coefficient range albeit with decreased peak values. The incorporation of negative capacitance significantly expands the low-frequency bandgap range. Visualized through vibration transfer simulations, the vibration-damping ability of the piezoelectric phononic crystal is demonstrated. Experimentally, this paper finds that two propagation modes of bending waves (symmetric and anti-symmetric) result in variable voltage amplitudes, and the average vibration of the system decreases by 4–5 dB within the range of 1710–1990 Hz. The comparison between experimental and model-generated data confirms the accuracy of the attenuation coefficient calculation model. This convergence between experimental and computational results emphasizes the validity and usefulness of the proposed model, and this paper provides theoretical support for the application of piezoelectric phononic crystals in the field of plate–shell vibration reduction.

## 1. Introduction

Vibration is an inherent phenomenon that occurs during the operation of mechanical systems. While beneficial vibrations can be harnessed for various purposes, detrimental vibrations may cause structural damage, disrupt system functionality, and significantly shorten the lifespan of mechanical systems. Thus, effective vibration control is paramount in ensuring the stability, safety, and operational comfort of mechanical structures.

Vibration isolation can be mainly divided into active vibration isolation and passive vibration isolation [1,2,3]. Active vibration isolation involves the use of closed-loop control systems, utilizing actuators and other power output components to counteract vibrations. In contrast, passive vibration isolation primarily relies on structural arrangements between the vibration source and the isolated object to attenuate vibration transmission. Phononic crystals, as an innovative type of passive vibration damping material, consist of periodic structures made from two or more media. These structures modulate elastic waves at specific frequencies, creating a frequency range known as the forbidden band or bandgap [4,5]. By employing a thoughtful structural design, phononic crystals can effectively damp vibrations within designated frequency bands, introducing a novel approach to passive vibration isolation. The bandgap range of traditional phononic crystals primarily depends on the unit size and material composition. Once the material and structure are set in actual production, the elastic wave bandgap remains fixed. Furthermore, traditional phononic crystals require substantial additional mass, making it difficult to modify the original structure or to use them in low-strength structures, limiting their practical applicability. Consequently, novel metamaterials like piezoelectric phononic crystals are attracting increasing attention from researchers.

Piezoelectric phononic crystals utilize piezoelectric materials as scatterers. These smart materials can convert mechanical energy into electrical energy and dissipate it through shunt damping in external circuits [6,7]. This process modulates elastic waves via electrical resonance and active control without altering the original structure [8,9]. Li Zhengyang [10] systematically summarized the current research status of smart piezoelectric materials and classified them into single-type piezoelectric phononic crystals and metamaterials, embedded piezoelectric/elastic composite-type piezoelectric phononic crystals and metamaterials, and composite piezoelectric phononic crystals and metamaterials consisting of externally attached piezoelectric sheets and elastic structures. Patch-type piezoelectric sheets have a small additional mass, facilitating the implementation of external circuits and active control methods. Yi [11] categorized piezoelectric phononic crystals into shunt-damped and sensing-actuated types based on their working principles. The shunt-damped type utilizes the intrinsic capacitance of the piezoelectric sheet to create an electrical resonance effect with external circuits, thereby maximizing the attenuation of elastic waves. Wang [12] derived the transfer function of the piezoelectric phononic crystal semi-active control strategy using a digital control circuit and proposed a “pole-zero” control strategy; Sheng-Bing [13] used an anti-symmetric arrangement of piezoelectric sheets to control the wave propagation and attenuation characteristics of flexible beams and explored the changes in their vibration isolation performance.

Researchers globally have extensively studied piezoelectric phononic crystals. Shunt-damped piezoelectric phononic crystals use shunt circuit damping as the primary vibration isolation mechanism with the bandgap range and attenuation capability within the bandgap serving as key metrics for assessing their vibration isolation performance. Currently, commonly employed bandgap calculation methods include the Transfer Matrix Method [14], Spectral Element Method, Plane Wave Expansion Method [15], and Finite Element Method [16,17,18,19]. Kargozarfard [20] proposed an enhanced plane-wave expansion method that addresses the limitations of conventional plane-wave techniques and enables precise control of the bandgap structure via spatiotemporal modulation characteristics. Shen [21] utilized the “negative equivalent elastic modulus” property of piezoelectric sheets to approximate the local resonance bandgap range and verified it numerically. Panxue [22] derived the vibration transmission rate of electromechanical coupled systems under finite periods based on Timoshenko Beam Theory and investigated the effects of structural and electrical parameters on vibration damping performance. Zhiwen [23] developed a dispersion calculation model for piezoelectric phononic crystal plates using Mindlin’s theory and the plane wave expansion method, calculating shear correction coefficients via transcendental equations. Sugino [24] formulated the governing equations of a two-dimensional fully coupled electromechanical system and deduced the limiting-state form of the edge frequencies of the bandgap. Shengbing [25,26] created an acoustic transmission loss coefficient model based on dynamic equivalence theory. Milić [27] introduced a finite element analysis method founded on NURBS and Reissner–Mindlin kinematics to address the behavior of active composite laminates exhibiting electro-mechanical coupling effects. Nestorović [28] proposed an ABAQUS–Python co-simulation method that efficiently handles complex thin-walled structures and mitigates the locking effect. Since piezoelectric phononic crystals encompass both mechanical and electrical fields, coupling multi-physical fields is essential for simulation calculations. Analyzing the attenuation coefficient within the bandgap is crucial due to the damping properties of the external shunt circuit. Thus, it is necessary to analyze the piezoelectric phononic crystal using complex band theory. Current bandgap simulations for piezoelectric phononic crystals primarily rely on the Kirchhoff plate assumption to derive finite element models, which involve extensive mathematical operations and present challenges with solving eigenvalue functions.

In summary, this paper derives the formula for the complex band calculation of piezoelectric phononic crystals based on the equilibrium differential equation under plane stress conditions and the intrinsic equation of the piezoelectric sheet. By introducing the Bloch boundary condition through parameter changes, it utilizes the strong coupling capability of the mathematical partial differential equation (PDE) module to quickly calculate the bandgap range and attenuation coefficient magnitude within the bandgap. The obtained bandgap range is verified through the multi-physics coupling of COMSOL’s structural mechanics and electrical modules. The effects of different inductance, resistance, and capacitance parameters on the bandgap range and attenuation coefficient in the bandgap are analyzed for various coverage and external shunt circuits, and the resulting influence laws are summarized. The impact of electrical parameters on vibration isolation performance is validated by comparing the simulation of vibration transfer curves from finite element software with experimental results.

The research demonstrates that the complex energy band calculation method utilized in this paper exhibits excellent computability for determining the bandgap position, range, and attenuation coefficient in piezoelectric phonon crystals. Furthermore, the modeling with COMSOL finite element simulation software enhances the calculation method’s applicability to piezoelectric phonon crystals on complex patch surfaces, such as acoustic black hole surfaces. It is proven that piezoelectric phononic crystals exhibit excellent vibration damping performance under finite period arrangements. Through experiments, we analyze the signals generated by piezoelectric plates in different connection modes and investigate their underlying causes. The accuracy of the attenuation coefficient is validated by comparing it with the vibration transmission curve measured in experiments.

## 2. Structural Modeling and Calculation Methods

### 2.1. Calculated Parameters for the Complex Band of Piezoelectric Phononic Crystals

**Figure 1:**
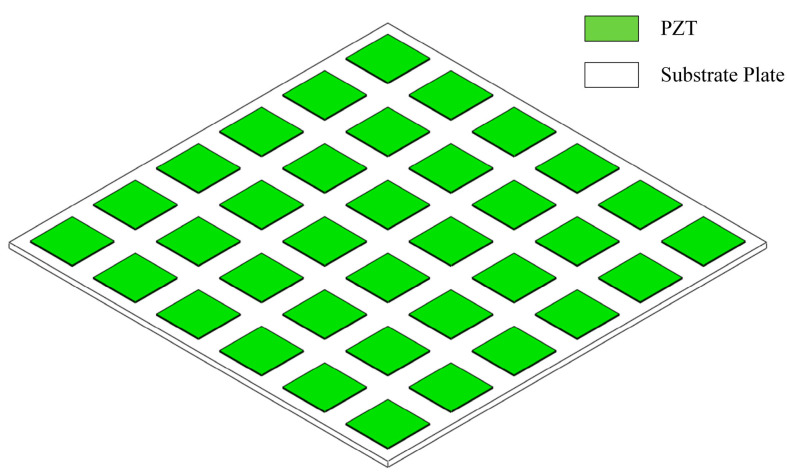
Piezoelectric phononic crystal plate.

**Figure 2:**
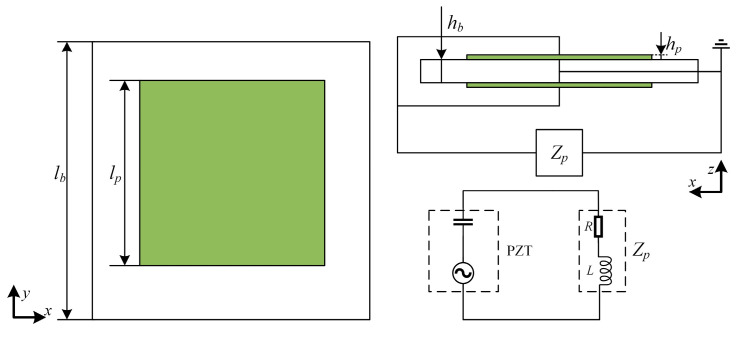
Schematic diagram of the unit cell.

As illustrated in Figure 1, the 2D piezoelectric phononic crystal plate primarily consists of a substrate plate, a piezoelectric ceramic sheet (PZT), and an external shunt circuit. Figure 2 shows the schematic diagram of its unit cell, where a thin plate functions as the substrate, and the piezoelectric sheet along with the external shunt circuit constitutes the scatterer. The piezoelectric ceramic sheet is symmetrically attached to the upper and lower surfaces of the substrate plate and is simultaneously connected to the external circuit. The width and thickness of the substrate plate for a single unit cell are denoted by *l_b_* and *h_b_*, respectively, while those of the piezoelectric sheet are denoted *l_p_* and *h_p_*. The complex impedance of the external shunt circuit is denoted by *Z_p_*.

In the force–electric coupling system of piezoelectric phononic crystals, the piezoelectric sheet, as a smart material, can convert mechanical energy into electrical energy. When the substrate plate vibrates at an angular frequency *ω* with simple harmonic waves, the piezoelectric sheet on its upper and lower surfaces uses the positive piezoelectric effect to convert mechanical energy into electrical energy. This energy is dissipated through the external shunt damping circuit. According to the structural properties of the phononic crystal, which possesses a two-dimensional periodic structure, the displacement field can be decomposed by applying Bloch’s theorem as follows:

(1) $$u\left(r,t\right)=u_{k}\left(r\right)e^{i\left(k\cdot r-\omega t\right)}$$

For analyzing real bands in COMSOL’s structural mechanics mode, the coupling between multiphysics fields is utilized by setting up structural mechanics, electrostatics, and circuit modules. The piezoelectric effect facilitates state coupling between the mechanical and electrical fields. By scanning the wavevector ***k***, different eigenmodes of the structure are obtained. When no eigenmode exists in a corresponding interval, this interval is identified as the bandgap. This method is known as real band analysis or *ω*(***k***)-analysis. In this method, only real numbers are used for scanning the wavevector ***k***. This allows for determining the position of the bandgap in the energy band analysis, but it does not provide the specific attenuation coefficient within the bandgap. For piezoelectric phononic crystals under force-electrical coupling, real band analysis cannot reflect the width of the local resonance bandgap or the damping effect in the shunt circuit, only the center of the local resonance bandgap. Consequently, real band analysis cannot determine the range of the local resonance bandgap or the attenuation capability of elastic waves within this range.

Obtaining the eigenwave vector ***k*** by specifying the angular frequency *ω* is known as complex band analysis or ***k***(*ω*) analysis. This method yields a complex expression of the eigenwave vector:

(2) $$u\left(r,t\right)=u_{k}\left(r\right)e^{i\left[\left(k_{r}+ik_{i}\right)\cdot r-\omega t\right]}=u_{k}\left(r\right)e^{i\left(k_{r}\cdot r-\omega t\right)}\times e^{-k_{i}}$$

where *k_r_* and *k_i_* represent the real and imaginary parts of the wave vector, respectively. Compared to real band analysis, this method introduces the imaginary part of the wave vector to describe the amplitude changes in the vibration of the elastic wave. For a force–electric coupled system analyzed using complex bands, the intrinsic wave vector ***k*** obtained by scanning the angular frequency can reflect the range of the local resonance bandgap due to circuit resonance as well as the magnitude of the attenuation coefficient within the bandgap.

For the piezoelectric phononic crystal analyzed in this paper, the thicknesses of the substrate plate and the piezoelectric sheet are much smaller than the minimum size of the midplane. Therefore, the analysis can be simplified using thin-plate theory:

(3) $$\begin{array}{ccc} \left\{\begin{array}{c} \sigma_{x}=\frac{Y}{1-\mu^{2}}(\varepsilon_{x}+\mu \varepsilon_{y})\\ \sigma_{y}=\frac{Y}{1-\mu^{2}}(\varepsilon_{y}+\mu \varepsilon_{x})\\ \tau_{xy}=\frac{Y}{2\left(1+\mu \right)}\gamma_{xy} \end{array}\right. & & \left\{\begin{array}{r} \varepsilon_{x}=\frac{\partial u_{1}}{\partial x}\\ \varepsilon_{y}=\frac{\partial u_{2}}{\partial y}\\ \gamma_{xy}=\frac{\partial u_{2}}{\partial x}+\frac{\partial u_{1}}{\partial y} \end{array}\right. \end{array}$$

where *Y* is the Young’s modulus of the substrate plate, μ is the Poisson’s ratio of the substrate plate, *u*_1_ is the displacement component in the *x*-direction, and *u*_2_ is the displacement component in the *y*-direction. The equilibrium differential equation of any point in the plane stress state is

(4) $$\left\{\begin{array}{l} \frac{\partial \sigma_{x}}{\partial x}+\frac{\partial \tau_{xy}}{\partial y}+f_{x}=m{\ddot{u}}_{1}\\ \frac{\partial \sigma_{y}}{\partial y}+\frac{\partial \tau_{xy}}{\partial x}+f_{y}=m{\ddot{u}}_{2} \end{array}\right.$$

Substituting Equation (3) into (4) yields

(5) $$\begin{array}{c} \left\{\frac{\partial }{\partial x}\left[\frac{Y}{1-\mu^{2}}\frac{\partial u_{1}}{\partial x}+\frac{Y\mu }{1-\mu^{2}}\frac{\partial u_{2}}{\partial y}\right]+\frac{\partial }{\partial y}\left[\frac{Y}{2\left(1+\mu \right)}\frac{\partial u_{2}}{\partial x}+\frac{Y}{2\left(1+\mu \right)}\frac{\partial u_{1}}{\partial y}\right]\right\}-\rho \frac{\partial^{2}u_{1}}{\partial t^{2}}=-f_{x}\\ \left\{\frac{\partial }{\partial y}\left[\frac{Y}{1-\mu^{2}}\frac{\partial u_{2}}{\partial y}+\frac{Y\mu }{1-\mu^{2}}\frac{\partial u_{1}}{\partial x}\right]+\frac{\partial }{\partial x}\left[\frac{Y}{2\left(1+\mu \right)}\frac{\partial u_{2}}{\partial x}+\frac{Y}{2\left(1+\mu \right)}\frac{\partial u_{1}}{\partial y}\right]\right\}-\rho \frac{\partial^{2}u_{2}}{\partial t^{2}}=-f_{y} \end{array}$$

By introducing a parametric transformation equation, the Bloch boundary condition is converted into a general continuum periodic boundary condition. This transformation eliminates the effect of the scanning dependent variable, specifically the intrinsic wave vector, in the boundary condition. The displacement field parametric transformation equation can be expressed as [29]

(6) $$u\left(r,t\right)=u_{k}\left(r\right)e^{ik\left(\cos\theta x+\sin\theta y\right)-i\omega t}$$

The parametrically transformed displacement field is substituted into Equation (5) and simplified, as detailed in Appendix A. The piezoelectric sheets are polarized along the *z*-axis with the assumption that they are uniformly polarized. The dimension in the polarization direction, i.e., the thickness of the piezoelectric sheet, is significantly smaller than its length and width. The first type of piezoelectric equation system can be expressed as

(7) $$\left[\begin{array}{c} S_{1}\\ S_{2}\\ S_{6}\\ D_{3} \end{array}\right]=\left[\begin{array}{cccc} s_{11}^{E} & s_{12}^{E} & 0 & d_{31}\\ s_{12}^{E} & s_{11}^{E} & 0 & d_{31}\\ 0 & 0 & s_{66}^{E} & 0\\ d_{31} & d_{31} & 0 & \varepsilon_{33}^{T} \end{array}\right]\left[\begin{array}{c} T_{1}\\ T_{2}\\ T_{6}\\ E_{3} \end{array}\right]$$

where $s_{11}^{E}$, $s_{12}^{E}$, and $s_{66}^{E}$ represent the flexibility coefficient of the piezoelectric sheet under constant electric field strength, *d*_31_ represents the piezoelectric constant, and $\varepsilon_{33}^{T}$ represents the dielectric constant under constant stress.

Assuming the external excitation wavelength is much larger than the length and width of the piezoelectric sheet, placing the piezoelectric sheet within the approximate wavelength range, the electric field strength *E*_3_ can be considered uniform throughout. As shown in Figure 2, the external circuit connection can be modeled as an AC power supply and capacitance. The upper and lower surfaces of the piezoelectric sheet are connected in parallel, and the voltage across the two terminals of the piezoelectric sheet is equal to the superimposed charge. Based on the external circuit connection and the voltage–current relationship, the following can be derived:

(8) $$I=-2\times sQ_{p}=\frac{U}{Z_{p}}$$

and

(9) $$\begin{array}{ccc} s=i\omega & U=\int_{\Omega }E_{3}dz=E_{3}h_{p} & Q_{p}=\iint_{\Omega }D_{3}dxdy=A_{p}D_{3} \end{array}$$

The combinations yield

(10) $$D_{3}=-\frac{h_{p}}{2\times sZA_{p}}E_{3}$$

Substituting the equation for the potential shift in terms of electric field strength into the first type of piezoelectric system equations yields the following:

(11) $$E_{3}=-\frac{2\times d_{31}A_{p}}{h_{p}}\times \frac{1}{\frac{1}{sZ}+2\times C_{p}^{T}}\left(T_{1}+T_{2}\right)$$

where $C_{p}^{T}=\varepsilon_{33}^{T}A_{p}/h_{p}$ is the intrinsic capacitance of the piezoelectric sheet under mechanically free boundary conditions, corresponding to the constant stress state. To analyze the local resonance bandgap generation mechanism of piezoelectric phononic crystals under a force–electric coupling system, it is essential to consider the intrinsic capacitance formed by the piezoelectric sheet and the inductance of the external circuit, which together form an electrical resonance under an AC source. Therefore, studying the intrinsic capacitance of the piezoelectric sheet is crucial for determining the center band of the local resonance bandgap theoretically. The capacitance of the piezoelectric sheet is dynamic during operation, and it is always situated between the two limiting states: the completely free and completely cut-off states. Consequently, its dynamic capacitance lies between the capacitance of the constant stress state and the constant strain state. The boundary conditions determined by the first type of piezoelectric equations represent the completely free boundary in the constant stress state. Similarly, the complete cut-off state in the constant strain state is calculated by the third type of piezoelectric equations. The piezoelectric sheet capacitance in these states is given by

(12) $$C_{p}^{S}=\frac{\varepsilon_{33}^{T}A_{p}}{h_{p}}\left(1-\frac{2s_{11}^{E}}{s_{11}^{E}+s_{12}^{E}}\times \frac{d_{31}^{2}}{s_{11}^{E}\varepsilon_{33}^{T}}\right)$$

The dynamic capacitance of the piezoelectric sheet always lies within the two limit state intervals. The presence of dynamic capacitance during operation can be corrected for the local resonance bandgap center position by introducing a capacitance correction factor τ. By substituting the electric field strength into the stress–strain equations for coupled mechanical and electrical field modeling, the first type of piezoelectric equation set can be transformed into

(13) $$\left\{\begin{array}{l} T_{1}=\frac{s_{11}^{E^{\prime }}}{{\left(s_{11}^{E^{\prime }}\right)}^{2}-{\left(s_{12}^{E^{\prime }}\right)}^{2}}S_{1}+\frac{-s_{12}^{E^{\prime }}}{{\left(s_{11}^{E^{\prime }}\right)}^{2}-{\left(s_{12}^{E^{\prime }}\right)}^{2}}S_{2}\\ T_{2}=\frac{-s_{12}^{E^{\prime }}}{{\left(s_{11}^{E^{\prime }}\right)}^{2}-{\left(s_{12}^{E^{\prime }}\right)}^{2}}S_{1}+\frac{s_{11}^{E^{\prime }}}{{\left(s_{11}^{E^{\prime }}\right)}^{2}-{\left(s_{12}^{E^{\prime }}\right)}^{2}}S_{2}\\ T_{6}=\frac{1}{s_{66}^{E}}S_{6} \end{array}\right.$$

where

(14) $$s_{11}^{E^{\prime }}=s_{11}^{E}-\frac{2\times d_{31}A_{p}sZ}{h_{p}\left(1+2\times sZ\tau C_{p}^{T}\right)}$$

(15) $$s_{12}^{E^{\prime }}=s_{12}^{E}-\frac{2\times d_{31}A_{p}sZ}{h_{p}\left(1+2\times sZ\tau C_{p}^{T}\right)}$$

Similarly, the control differential equations in the piezoelectric domain can be obtained by substituting the parameter-transformed displacement field expressions. These equations can then be combined with the control differential equations in the substrate plate domain. For the force–electrically coupled piezoelectric phononic crystal, the COMSOL PDE module can be used to quickly solve the second-order partial differential equations and impose general continuity conditions. The overall control equations can be listed as follows:

(16) $$\lambda^{2}e_{a}U+\nabla \cdot \left(-c\nabla U-\alpha U\right)+\beta \cdot \nabla U+aU=F$$

where

$$\begin{array}{c} \begin{array}{cc} \begin{array}{ccc} \nabla =\left[\begin{array}{cc} \frac{\partial }{\partial x} & \frac{\partial }{\partial y} \end{array}\right] & U=\left[\begin{array}{c} u_{1}\\ u_{2} \end{array}\right] & F=\left[\begin{array}{c} F_{Sx}\\ F_{Sy} \end{array}\right] \end{array} & \begin{array}{cc} a=\left[\begin{array}{cc} -\rho \omega^{2} & 0\\ 0 & -\rho \omega^{2} \end{array}\right] & \lambda =ik \end{array} \end{array}\\ \begin{array}{cc} e_{a}=\left[\begin{array}{cc} -n_{1}\cos^{2}\theta -n_{3}\sin^{2}\theta & -\left(n_{2}+n_{3}\right)\sin\theta \cos\theta \\ -\left(n_{2}+n_{3}\right)\sin\theta \cos\theta & -n_{1}\sin^{2}\theta -n_{3}\cos^{2}\theta \end{array}\right] & \beta =\left[\begin{array}{cc} \left(\begin{array}{c} -n_{1}\cos\theta \left(ik\right)\\ -n_{3}\sin\theta \left(ik\right) \end{array}\right) & \left(\begin{array}{c} -n_{3}\sin\theta \left(ik\right)\\ -n_{2}\cos\theta \left(ik\right) \end{array}\right)\\ \left(\begin{array}{c} -n_{2}\sin\theta \left(ik\right)\\ -n_{3}\cos\theta \left(ik\right) \end{array}\right) & \left(\begin{array}{c} -n_{3}\cos\theta \left(ik\right)\\ -n_{1}\sin\theta \left(ik\right) \end{array}\right) \end{array}\right] \end{array}\\ \begin{array}{cc} C=\left[\begin{array}{cc} \left(\begin{array}{cc} n_{1} & 0\\ 0 & n_{3} \end{array}\right) & \left(\begin{array}{cc} 0 & n_{2}\\ n_{3} & 0 \end{array}\right)\\ \left(\begin{array}{cc} 0 & n_{3}\\ n_{2} & 0 \end{array}\right) & \left(\begin{array}{cc} n_{3} & 0\\ 0 & n_{1} \end{array}\right) \end{array}\right] & \alpha =\left[\begin{array}{cc} \left(\begin{array}{c} n_{1}\cos\theta \left(ik\right)\\ n_{3}\sin\theta \left(ik\right) \end{array}\right) & \left(\begin{array}{c} n_{2}\sin\theta \left(ik\right)\\ n_{3}\cos\theta \left(ik\right) \end{array}\right)\\ \left(\begin{array}{c} n_{3}\sin\theta \left(ik\right)\\ n_{2}\cos\theta \left(ik\right) \end{array}\right) & \left(\begin{array}{c} n_{3}\cos\theta \left(ik\right)\\ n_{1}\sin\theta \left(ik\right) \end{array}\right) \end{array}\right] \end{array} \end{array}$$

For substrate panels:

$$\begin{array}{ccc} n_{1}=\frac{Y_{b}}{1-{\mu_{b}}^{2}} & n_{2}=\frac{Y_{b}\mu_{b}}{1-{\mu_{b}}^{2}} & n_{3}=\frac{Y_{b}}{2\left(1+\mu_{b}\right)} \end{array}$$

For piezoelectric sheets:

$$\begin{array}{ccc} n_{1}=\frac{s_{11}^{E^{\prime }}}{{\left(s_{11}^{E^{\prime }}\right)}^{2}-{\left(s_{11}^{E^{\prime }}\right)}^{2}} & n_{2}=\frac{-s_{12}^{E^{\prime }}}{{\left(s_{11}^{E^{\prime }}\right)}^{2}-{\left(s_{11}^{E^{\prime }}\right)}^{2}} & n_{3}=\frac{1}{s_{66}^{E}} \end{array}$$

### 2.2. Model Validation

In this paper, the COMSOL solid mechanics, electrostatics, and circuits modules are used to perform real band calculations for piezoelectric phononic crystals through the piezoelectric effect coupled with multiphysics fields. The substrate plate is made of epoxy resin, and the piezoelectric sheet model is PZT-5H. The specific material parameters are shown in Table 1 and Table 2.

**Figure 3:**
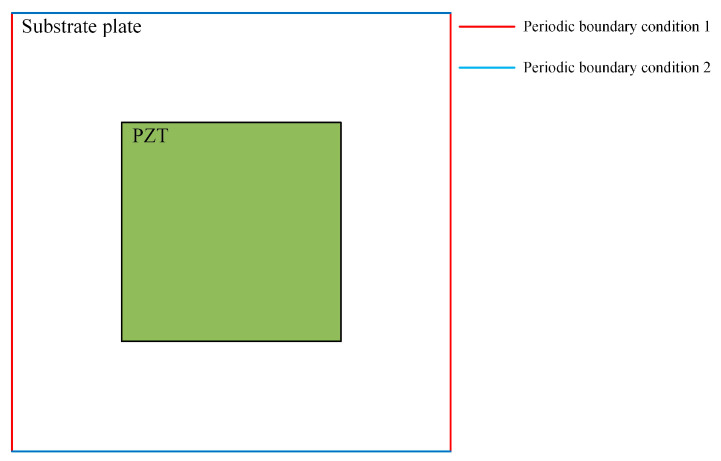
Imposed boundary condition.

The external shunt circuit employs an LR shunt damping circuit, integrating structural mechanics with the electrical module. Periodic boundary conditions are implemented through structural mechanics, as illustrated in Figure 3. The circuit terminal is positioned on the upper and lower surfaces of the piezoelectric plate via the electrostatic module, and it is coupled with the electrical device through the circuit module to resolve the real band.

The solution process includes some in-plane modes, but the generation of positive and negative piezoelectric effects and the realization of vibration isolation occur in the out-of-plane modes. Since the piezoelectric phononic crystal provides vibration isolation for bending waves (out-of-plane modes), it is necessary to establish a screening criterion for bending waves and exclude the in-plane modes [30]:

(17) $$\beta \_Modal=\frac{{∭}_{V}ww^{*}dxdydz}{{∭}_{V}\left(uu^{*}+vv^{*}+ww^{*}\right)dxdydz}$$

where, *u*, *v*, and *w*, respectively, represent the displacement of the body element at any point in the *x*, *y*, and *z* direction; u^*^, v^*^, and w^*^, respectively, represent the body element at any point in the direction of the displacement of the conjugate complex of the *x*, *y*, *z* directions, taking *β_Modal* greater than or equal to 0.9 as the out-of-plane mode.

**Figure 4:**
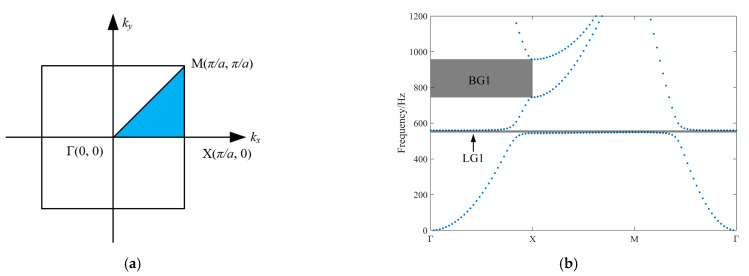
Real band diagram of piezoelectric phonon crystal. (**a**) Diagram of the 2D irreducible Brillouin zone. (**b**) Real band curve (shaded area represents the bandgap range).

As shown in Figure 4a, due to the spatial symmetry of the lattice structure of phonon crystals, for two-dimensional plane problems, the band curve can be obtained by simply scanning the wave vector ***k*** along the boundary of the minimum irreducible Brillouin zone, as illustrated in Figure 4b. Two bandgap ranges are generated in the ΓX direction. The first bandgap (LG1, 581–590 Hz) is dominated by the local resonance effect, resulting from the electrical resonance between the intrinsic capacitance of the piezoelectric sheet and the inductance of the external circuit. This bandgap is extremely narrow, because real band analysis cannot reflect the resistive damping effects of the piezoelectric phononic crystal. The second bandgap (BG1, 730–990 Hz) is dominated by the Bragg scattering effect, where the elastic wave is confined by the periodic medium, resulting in attenuation. The MΓ direction generates a bandgap identical in mechanism and position to the first bandgap in the ΓX direction. This is mainly because the local resonance bandgap formed by electrical resonance is not directionally selective and can form an omnidirectional bandgap.

Because the real band cannot reflect the impact of resistive damping, the resistance R is set to 0 Ω during the complex band analysis to maintain consistency between the real and complex bands. For the complex band analysis, the elastodynamic fluctuation equations and the electric field are coupled through the COMSOL PDE module using second-order partial differential equations. The wave equation is input in the form of Equation (16), and the boundary is constrained by Dirichlet boundary conditions (general continuity boundaries).

(18) $$\begin{array}{c} u_{dst}=u_{src}\\ -n_{dst}\cdot {\left(-c\nabla U-\alpha U\right)}_{i,dst}=-n_{src}\cdot {\left(-c\nabla U-\alpha U\right)}_{i,src}\\ \begin{array}{cc} u=\left[\begin{array}{c} u_{1}\\ u_{2} \end{array}\right] & i=\left[\begin{array}{c} 1\\ 2 \end{array}\right] \end{array} \end{array}$$

Similarly, the in-plane modes are screened out by data screening, and the eigenwave vectors of the out-of-plane modes are solved for *k*. When the eigenwave vectors are all real, the wave can propagate without attenuation. When the eigenwave vectors contain an imaginary part, it indicates that the wave will attenuate during transmission. The propagation coefficient is defined as

**Figure 5:**
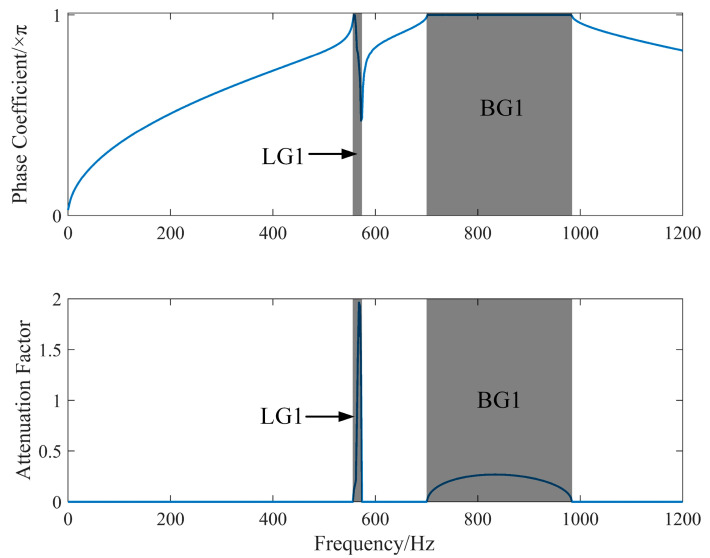
Complex band diagram of piezoelectric phonon crystal (ΓX).

(19) η=−ika

where *a* is the lattice constant. The real part represents the phase change in the wave vector, and the imaginary part indicates the magnitude of the wave attenuation coefficient during propagation. The phase coefficient changes abruptly within the local resonance bandgap, while the phase constant remains unchanged within the Bragg scattering bandgap. By comparing the real band with the center of the local resonance bandgap of the complex band, the dynamic capacitance correction coefficient is determined to be 0.85. The corrected complex band curve is plotted as shown in Figure 5.

As shown in Figure 5, the phase coefficients in the 0° complex band curves calculated by the PDE module exhibit a high degree of similarity with the real band curves obtained from ΓX directional scanning using the structural mechanics coupling module. This consistency verifies the accuracy of the complex band calculation method used in this paper. Since the local resonance bandgap generated by piezoelectric sheet force–electric coupling is not directionally selective, and this paper focuses on the local resonance bandgap generated by force–electric coupling, the simulation of the PDE module in this paper is based on the local resonance bandgap at 0° to simplify the model calculation. The position of the local resonance bandgap and the decay coefficient inside the bandgap are analyzed under the condition of 0° directional scanning.

## 3. Analysis of the Influence Law of Structural/Electrical Parameters Based on Attenuation Coefficients

### 3.1. Effect of Coverage on Bandgap

According to the bandgap generation principle of piezoelectric phononic crystals and the series-parallel circuit resonance equation, the coverage is directly related to the position of the local resonance bandgap. The coverage is defined as

(20) $$\chi =\frac{l_{p}}{l_{b}}$$

In order to exclude the influence produced by the Bragg bandgap, this paper selects the substrate plate material as the aluminum plate material close to the elastic modulus and Poisson’s ratio of PZT-5H, the lattice constant (width of the substrate plate, *l_b_*) is 80 mm, the thickness of the substrate plate, *h_b_*, is 4 mm, and the intrinsic capacitance of the piezoelectric sheet can be expressed in terms of the coverage ratio as

(21) $$\frac{2\times \varepsilon_{33}^{T}\chi^{2}A_{b}}{h_{p}}=2\varepsilon_{33}^{T}A_{b}\frac{\chi^{2}}{h_{p}}$$

The resonance center interval, which is the location of the center band of the local resonance bandgap, can be obtained as

(22) $$f_{\chi }=\frac{\sqrt{h_{p}}}{2\pi \sqrt{L\times 2\tau \varepsilon_{33}^{T}A_{b}}}\times \frac{1}{\chi }$$

**Figure 6:**
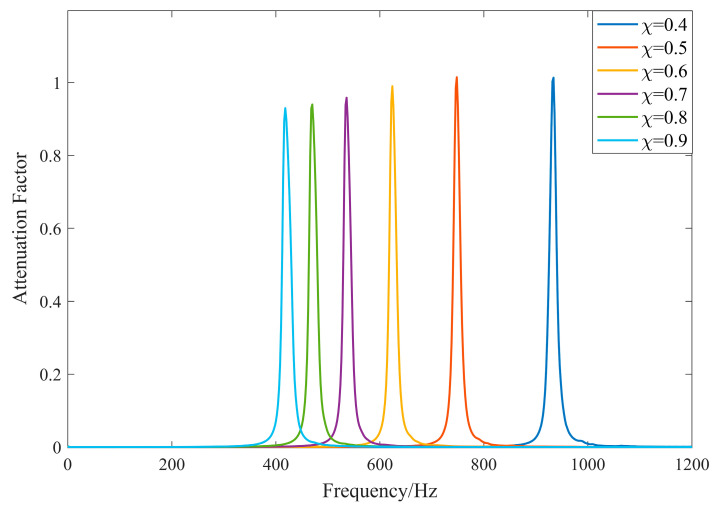
Attenuation coefficient for different coverage.

When the thickness of the piezoelectric sheet is fixed and used with the external shunt circuit, the resonance interval becomes a power exponential function of the coverage. The thickness of the piezoelectric sheet is set to 1 mm, the inductance of the external circuit is 0.6 H, the resistance is 30 Ω, and the coverage ranges from 0.4 to 0.9. The complex band attenuation coefficient is calculated at intervals of 0.1. The resulting attenuation coefficient curve is shown in Figure 6.

As shown in Figure 6, changes in coverage primarily affect the position of the center band of the local resonance bandgap with minimal impact on the attenuation coefficient in the forbidden band interval. The effect on the center band position is mainly due to the change in the intrinsic capacitance of the piezoelectric sheet caused by the variation in the coverage ratio, which leads to changes in the resonance interval. To further explore the effect of coverage and eliminate the influence of changes in the intrinsic capacitance of the piezoelectric sheet on the results, normalized coverage is used:

(23) $$\chi^{*}=\frac{l_{p}}{l_{b}}=\sqrt{\frac{h_{p}^{*}}{h_{p}}}$$

where $h_{p}^{*}$ is the thickness of the piezoelectric sheet after the change, and *h_p_* is 1 mm. At this time, the position of the center band of the local resonance bandgap is

(24) $$f_{\chi }=\frac{\sqrt{h_{p}}}{2\pi \sqrt{L\times 2\tau \varepsilon_{33}^{T}A_{b}}}$$

From Equation (24), after applying the normalized coverage setting, the position of the center band of the local resonance bandgap becomes a constant value, excluding the effect of the variation in the intrinsic capacitance of the piezoelectric sheet.

**Figure 7:**
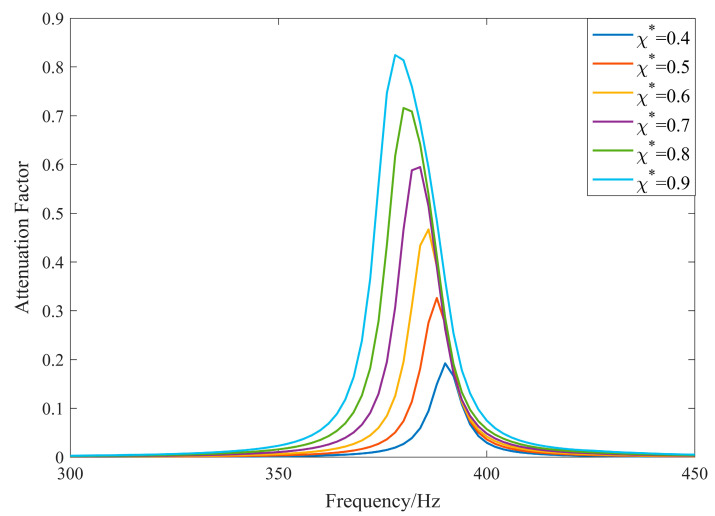
Attenuation coefficient for different normalized coverages.

As shown in Figure 7, the position of the center band of the local resonance bandgap is independent of the variation in normalized coverage. As derived from the theory, increasing the normalized coverage results in a wider bandgap and a higher attenuation coefficient within the bandgap.

When the area of the piezoelectric sheet changes but the thickness remains constant, the intrinsic capacitance of the piezoelectric sheet changes, causing the position of the center band of the local resonance bandgap to shift, exhibiting a power-exponential relationship with the coverage rate, while the attenuation coefficients within the forbidden band remain the same. When both the area and thickness of the piezoelectric sheet change, i.e., when the normalized coverage rate changes, the position of the center band of the local resonance bandgap remains unchanged, and the range of the bandgap and the attenuation coefficient within the bandgap increase with the increasing normalized coverage rate.

### 3.2. Effect of Inductance on Bandgap

**Figure 8:**
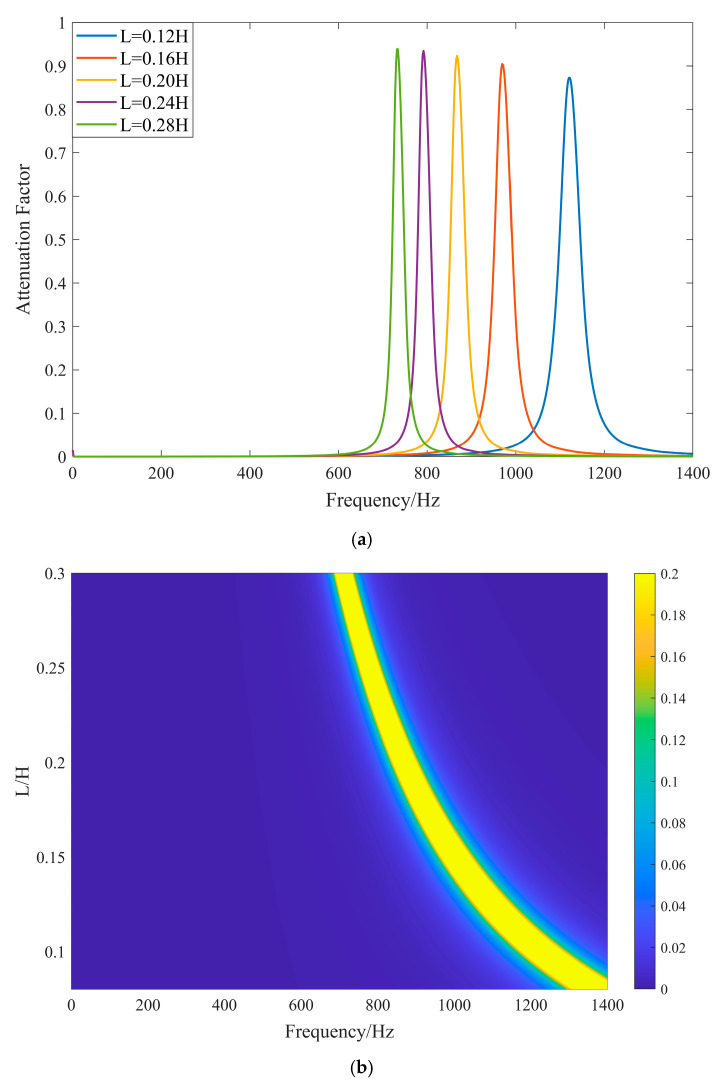
Attenuation coefficient with different inductances. (**a**) Comparison of discrete values for different inductances. (**b**) Comparison of continuous values for different inductances (intensity of the color indicates the magnitude of the attenuation coefficient).

According to Equation (24), the inductance is a power exponential function of the central band of the local resonance. To further investigate the effect of inductance size on the bandwidth of the elastic wave band and the attenuation coefficient, we choose the width of the substrate plate *l_b_* to be 80 mm, the thickness of the substrate plate *h_b_* to be 5 mm, the width of the piezoelectric sheet *l_p_* to be 60 mm, the thickness of the piezoelectric sheet *h_p_* to be 1 mm, and the external shunt circuit to be the LR circuit with a resistance of 30 Ω and an inductance range of 0.08–0.30 H. The attenuation coefficients for different inductances are plotted in Figure 8.

From Figure 8, the central band of the local resonance bandgap is a power exponential function of the inductance size. This is mainly because the change in inductance leads to a change in the electrical resonance frequency, thus shifting the central band of the bandgap, which is consistent with the theoretical analysis. As the inductance value increases, the range of the bandgap gradually narrows, and the attenuation coefficient within the bandgap increases slightly. However, the overall fluctuation is not significant, which is mainly because the quality factor of the series resonance circuit, $Q=\sqrt{L/C}/R$, increases with the inductance. The overall quality factor of the circuit increases with the inductance and the current:

(25) $$I=\frac{I_{\max}}{\sqrt{1+{\left[\frac{2Q(\omega -\omega_{0})}{\omega_{0}}\right]}^{2}}}$$

The current response is accelerated, the passband is narrowed, and the resistive energy dissipation interval is reduced, resulting in a narrower local resonance bandgap.

### 3.3. Effect of Resistance on Bandgap

From the previous section, it is clear that changes in resistance do not affect the location of the center band of the local resonance bandgap. However, the main vibration isolation unit of the system is damped vibration isolation, and the energy dissipation method is mainly related to the resistance. The inductance of the external circuit is set to 0.2 H, and the resistance is varied from 0 to 300 ohms, with the size parameters being the same as in the previous section. The system attenuation coefficient is plotted accordingly.

**Figure 9:**
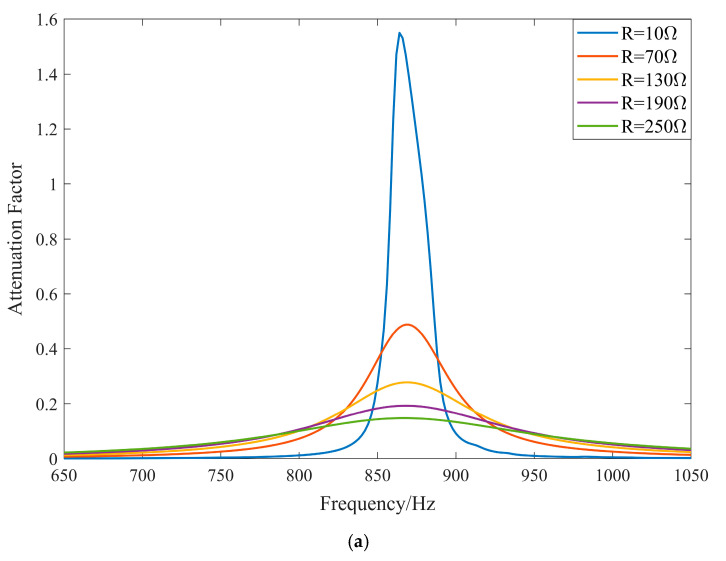

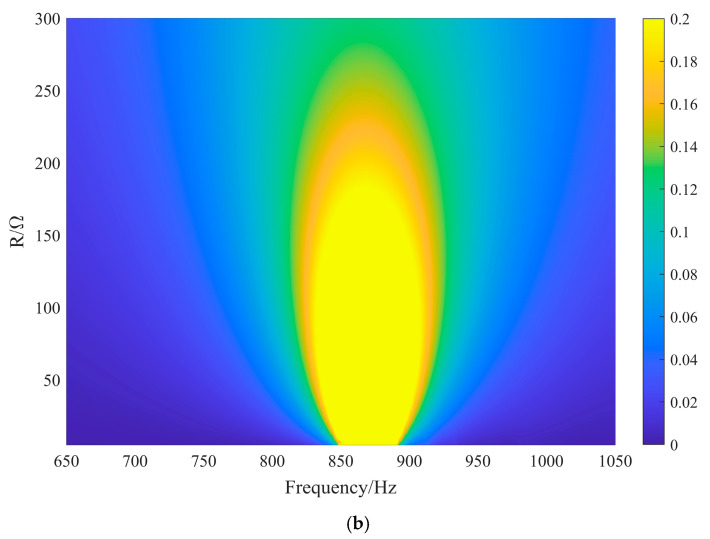
Attenuation coefficient with different resistances. (**a**) Comparison of discrete values for different resistances. (**b**) Comparison of continuous values for different resistances (intensity of the color indicates the magnitude of the attenuation coefficient).

From Figure 9, the larger the resistance, the wider the local resonance bandgap range, but the attenuation coefficient within the bandgap decreases. This is mainly because the circuit forms an electrical resonance with the intrinsic capacitance through the external circuit inductance and dissipates vibrational energy through the resistance. An increase in resistance results in a decrease in the peak current at resonance and a decrease in the peak attenuation coefficient within the bandgap. However, an increase in resistance can also significantly enhance the energy dissipation of the system, allowing it to dissipate energy at smaller currents, thereby widening the bandgap range.

### 3.4. Effect of Generalized Capacitance on Bandgap

The electromechanical coupling coefficient is the geometric mean of the reciprocal elastic dielectric energy density over the elastic self-energy density and the dielectric self-energy density, indicating the efficiency of conversion between mechanical and electrical energy. Limited by the electromechanical coupling coefficient, the width of the local resonance bandgap formed by the LR shunt circuit is narrow. According to the operating conditions and principles in the piezoelectric phononic crystal, the elastic self-energy density of the piezoelectric sheet is

(26) $$\begin{array}{ccc} U_{e}=\frac{1}{2}T_{h}s_{hk}^{E}T_{k} & & \left(h,k=1,2,3\right) \end{array}$$

The dielectric self-energy density is

(27) $$\begin{array}{ccc} U_{d}=\frac{1}{2}E_{i}\varepsilon_{ij}^{T}E_{j} & & \left(i,j=3\right) \end{array}$$

The mutually elastic dielectric energy density is

(28) $$\begin{array}{ccc} U_{m}=\frac{1}{2}E_{j}d_{jh}T_{h} & & \left(j=1,h=1,2,3\right) \end{array}$$

Its electromechanical coupling coefficient is

(29) $$k=\frac{U_{m}}{\sqrt{U_{e}U_{d}}}$$

According to the piezoelectric intrinsic equation, the electromechanical coupling coefficient of the system at this point is obtained:

(30) $$k_{31}=d_{31}\sqrt{\frac{1}{s_{11}^{E}\varepsilon_{33}^{T}}}=d_{31}\sqrt{\frac{2\times K_{a}}{C}}$$

where $K_{a}=A_{p}/\left(s_{11}^{E}h_{p}\right)$ represents the stiffness of the system when the piezoelectric elastomer is short-circuited, and *C* is the intrinsic capacitance of the piezoelectric double wafer. From Equation (30), the electromechanical coupling coefficient is related to the open-circuit stiffness and intrinsic capacitance of the piezoelectric sheet. The value of the electromechanical coupling coefficient generally ranges from 0.3 to 0.7. If the electromechanical coupling coefficient is too large, the system’s open-circuit stiffness can change considerably with the electrical boundary conditions, affecting the system’s stability. Improving the electromechanical coupling coefficient can be approached from two perspectives: open-circuit stiffness and intrinsic capacitance. When the system is connected in series with negative capacitance, according to the definition of the electromechanical coupling coefficient, it can be determined that

(31) $$k_{31}^{*}=d_{31}\sqrt{\frac{2\times K_{a}}{C^{*}}}$$

where C^*^ represents the system’s generalized capacitance. The series negative capacitance does not alter the original open-circuit stiffness and piezoelectric coefficient of the system; however, it modifies the generalized capacitance of the piezoelectric sheet within the system:

(32) $$k_{31}^{*}=k_{31}\times \sqrt{\frac{2-\alpha }{1-\alpha }}$$

where *α* represents the magnitude of the absolute value of the ratio between the negative capacitance and the intrinsic capacitance of the piezoelectric sheet. The introduction of the negative capacitance circuit alters the system’s generalized capacitance and thereby shifts the resonance position:

(33) $$f_{C^{*}}=\frac{1}{2\pi \sqrt{LC_{p}}}\times \sqrt{\frac{2-\alpha }{1-\alpha }}$$

The introduction of negative capacitance enhances the electromechanical coupling coefficient and reduces the system’s generalized capacitance, thereby effectively broadening the local resonance bandgap. However, this also impacts the position of the electrical resonance, shifting the local resonance bandgap toward higher frequencies.

**Figure 10:**
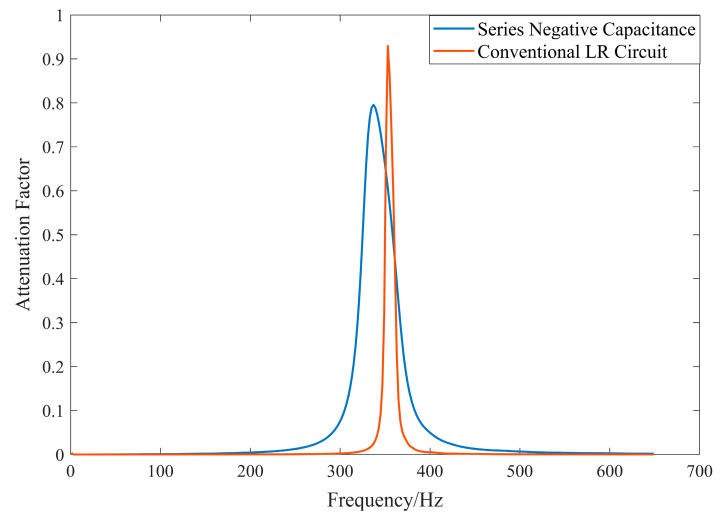
Comparison between negative capacitance circuits and traditional LR circuits.

From Figure 10, the introduction of negative capacitance significantly enhances the bandgap range compared to conventional circuits, owing to the improved electromechanical coupling coefficient. However, this necessitates a larger inductor to compensate for the reduced generalized capacitance, thereby decreasing the overall quality factor of the circuit and leading to the attenuation of the peak attenuation coefficient.

## 4. Vibration Transfer Simulation Analysis and Experiment

**Figure 11:**
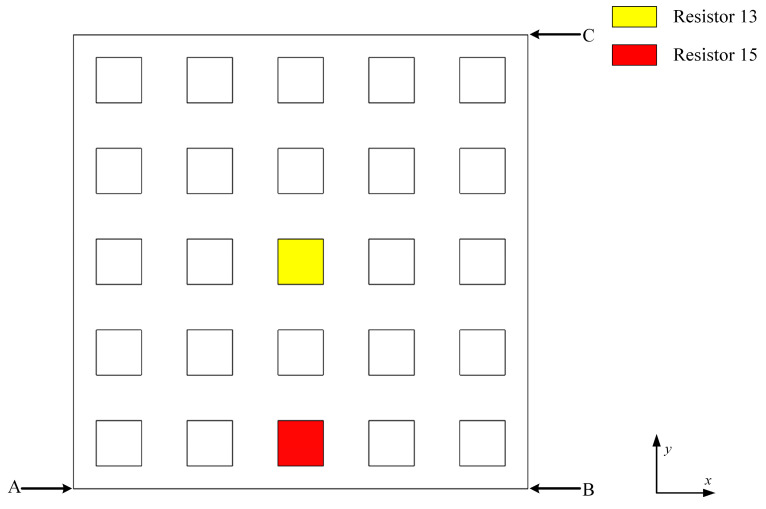
Finite element model.

A five-cycle piezoelectric phononic crystal plate is established, and a finite element model is constructed using COMSOL by coupling the structural mechanics, electrostatics, and circuits modules. The width of the substrate plate in the metacells is 60 mm with a thickness of 3.5 mm, while the piezoelectric sheet measures 40 mm in width and 1 mm in thickness. An LR external shunt circuit is employed, with an inductance of 0.1 H and a resistance of 300 Ω, as shown in Figure 11.

**Figure 12:**
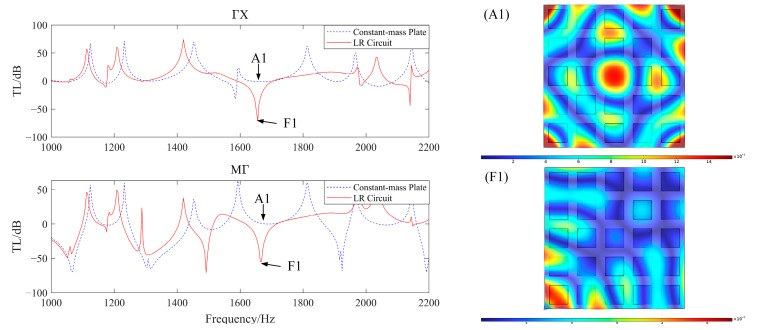
Vibration transfer curve (simulation).

The excitation point is positioned at point A. Acceleration response curves are recorded at point B in the 0° direction and at point C in the 45° direction, forming the transfer curve TL as follows:

(34) $$TL=20\times \log\left(\frac{a_{out}}{a_{in}}\right)$$

where *a_out_* is the output point acceleration and *a_in_* is the excitation point acceleration. Plotting the vibration transfer curve, theoretical calculations place the resonance position at 1736 Hz. As shown in Figure 12, the vibration transfer curve indicates that vibration attenuation occurs at this resonance position in both the 0° and 45° directions, thereby validating the theoretical analysis. Among these, the formation diagrams at points A1 and F1 visually illustrate the attenuation process of the bending wave.

**Figure 13:**
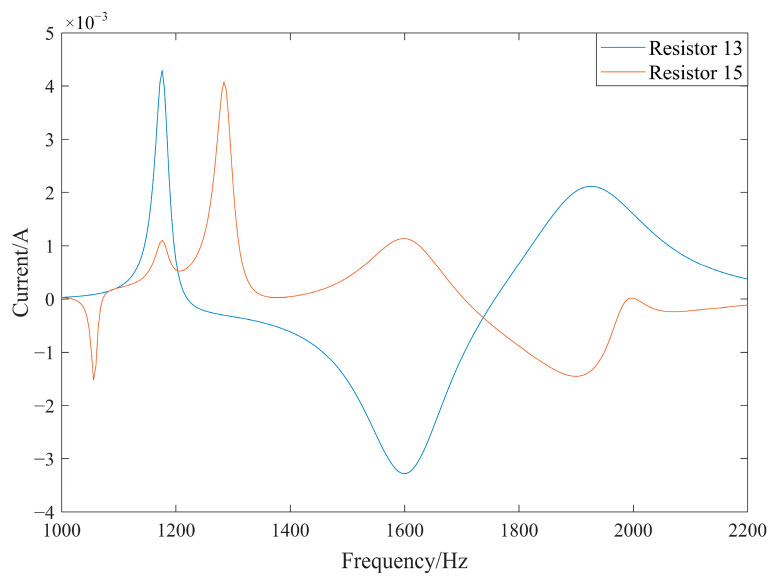
Current diagram (simulation).

The currents through resistors 13 and 15 during the simulation are depicted in Figure 13. Herein, the current peak due to the resonance effect is relatively flat, and resistors No. 13 and No. 15 exhibit the same current peak.

**Figure 14:**
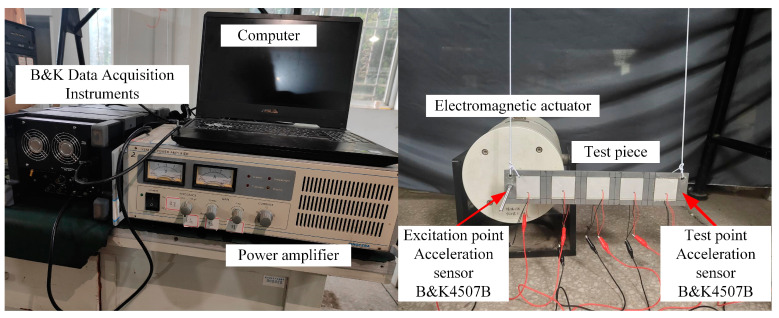
Experimental platform.

To verify the effectiveness of the piezoelectric phonon crystal in attenuating bending waves, an experimental platform was constructed, as shown in Figure 13. To reduce the complexity of the experiment, the 0° direction experimental curve, verified in the previous section, is used as the basis for analysis. This experiment employs a B&K data acquisition instrument to output the excitation signal, which is amplified by a power amplifier to drive the electromagnetic actuator. The acceleration signal is collected by a B&K4507B acceleration sensor and processed in the host computer for vibration signal analysis. The experimental test system constructed is depicted in Figure 14.

**Figure 15:**
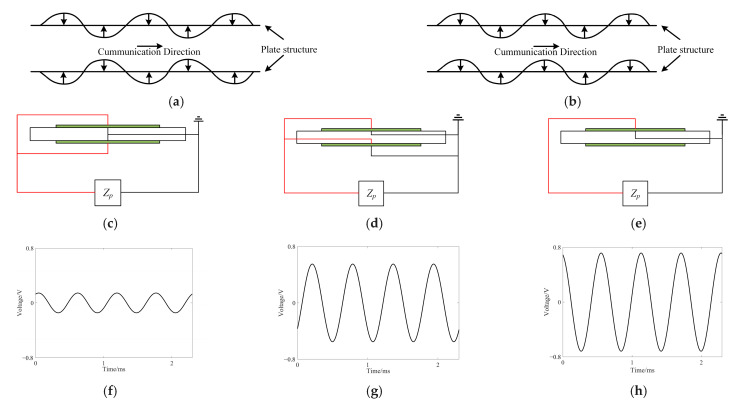
Forms of wave propagation and voltages for different connection forms. (**a**) Symmetrical model. (**b**) Anti-symmetrical model. (**c**) Positive/positive–negative/negative connection form (abbreviated as PPF). (**d**) Positive/negative–negative/positive connection form (abbreviated as PNF). (**e**) Form of unilateral connection (abbreviated as UCF). (**f**) Voltage measured by PPF. (**g**) Voltage measured by PNF. (**h**) Voltage measured by UCF.

This experiment employs a bilateral symmetric paste form. Theoretical analysis suggests that the piezoelectric sheet should produce the same voltage corresponding to the frequency of the excitation signal. However, in practice, the bending wave generated by the excitation process propagates in both symmetric and anti-symmetric modes. The propagation mode of the bending wave affects the amplitude of the voltage generated by different connection forms. The RIGOL DS110Z oscilloscope was employed to measure the voltage values generated by the piezoelectric sheet in various connection forms. The filtered data and their corresponding connection forms are presented below in Figure 15.

**Figure 16:**
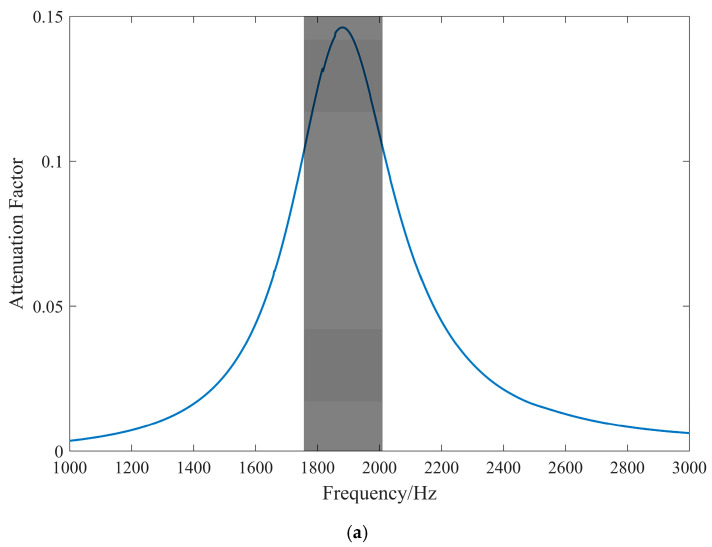

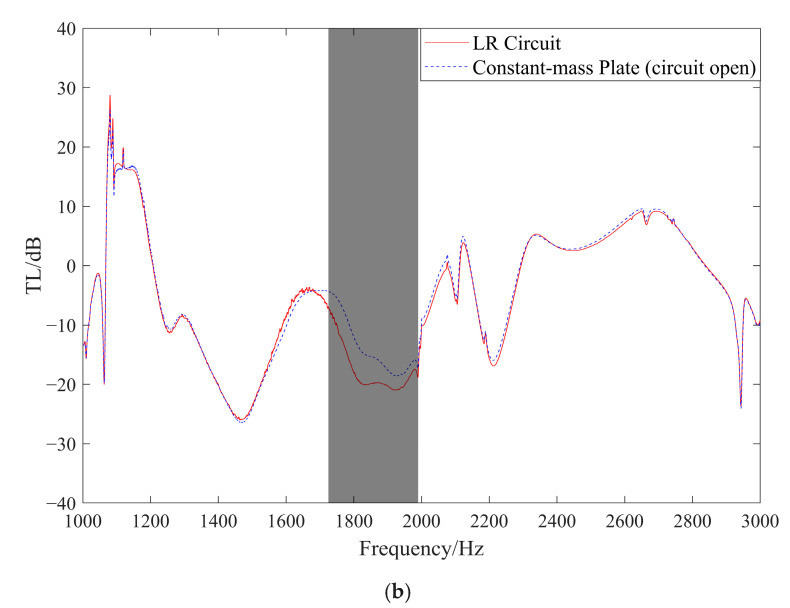
Comparison between simulation attenuation coefficient and experiment. (**a**) Attenuation factor (L = 0.1 H, R = 270 Ω). (**b**) Vibration transfer curves.

The frequency of the voltage generated by all three connection forms aligns with the frequency of the excitation source. However, the amplitude of the voltage produced by UCF is approximately the sum of the voltages produced by the PPF and PNF, as voltage can be produced in a unilateral mode regardless of whether the wave propagates in a symmetrical or anti-symmetrical mode. When the voltage amplitude is small, interference from the clutter signal dominates, resulting in a weak resonance phenomenon and drastically reduced vibration isolation effect. Thus, the PPF is not used in this experiment. The intrinsic capacitance of the piezoelectric sheet in the unilateral connection form is half that of the PNF, which is not conducive to reducing the resonant frequency. Therefore, this experiment employs the PNF. The external circuit uses a 0.1 H inductor, which has a larger DC internal resistance of approximately 270 Ω. In the 1000–3000 Hz frequency sweep, the attenuation coefficient and vibration transfer curve are shown in Figure 16 below.

The corresponding attenuation coefficient of the interval is 0.1–0.15, and the attenuation of the vibration amplitude after five cycles should be (e^−0.1^~e^−0.15^)^5^ times the original one, which is about 5–7 dB, corresponding to the experimentally measured attenuation of 5 dB and verifying the accuracy of the attenuation coefficient values.

## 5. Conclusions

In this paper, based on the plane stress state, energy band theory, and Bloch’s theorem, the partial differential equations of motion of the system under the influence of electrical parameters are derived using wave vector parameter transformations and numerical analysis. The complex band characteristic curves are solved, and the accuracy of the complex band model is verified by comparing them with the real band curves obtained from the multiphysics coupled field model.

Using the attenuation coefficient as an evaluation index, we analyze the effects of various structural and electrical parameters on the attenuation coefficient and examine the coupling influence of each parameter. By simulating vibration transmission, we can more intuitively observe the bending wave propagation process in various scenarios.

Experiments measured the voltage generated by the piezoelectric sheet under different connection forms and analyzed the reasons for its generation. The accuracy of the attenuation coefficient in the complex band model is verified by comparing it with the experimentally measured range and value of the vibration transfer curve.

In this work, we derive a complex energy band model for piezoelectric phononic crystals based on the equilibrium differential equations in the plane stress state using COMSOL PDE. We validate the model’s accuracy by comparing it with simulation and experimental data. This paper provides theoretical support for applying piezoelectric phononic crystals in plate–shell vibration reduction, especially for complex patch surfaces.

However, the complex band calculation method used in this paper requires scanning the wave vector directions of 0°, 45°, and 90° to obtain the complete band curve in the two-dimensional plane. Due to the extensive computational requirements, only the all-directional bandgap in the 0° direction is analyzed in this study, simplifying the design for future work. This approach enables quicker and more accurate acquisition of the complete energy band curve for piezoelectric phononic crystals in a two-dimensional plane.

## Figures and Tables

**Table 1:** Parameters of the substrate plate.

Name	Parameter
Width of a single unit cell *l_b_*/mm	80
Thickness of a single unit cell *h_b_*/mm	5
Density *ρ*/(kg/m^3^)	1180
Young’s modulus *Y_b_*/Pa	4.35 × 10^9^
Poisson’s ratio *μ_b_*	0.37

**Table 2:** Piezo sheet parameters.

Name	Parameter
Width of a single unit cell *l_p_*/mm	40
Thickness of a single unit cell *h_p_*/mm	0.2
Density *ρ*/(kg/m^3^)	7500
Flexibility coefficient $s_{11}^{E}$/(m^2^/N)	1.65 × 10^−11^
Flexibility coefficient $s_{11}^{E}$/(m^2^/N)	−4.78 × 10^−12^
Flexibility coefficient $s_{66}^{E}$/(m^2^/N)	4.26 × 10^−11^
Piezoelectric constant *d*_31_/(C/N)	−2.74 × 10^−10^
Dielectric constant $\varepsilon_{33}^{T}$/(F/m)	3.01 × 10^−8^

## Data Availability

The data used to support the findings of this study have been included within the article.

## Appendix A. Parameter Substitution Conversion Form (Base Plate)

$$\begin{array}{c} \begin{array}{l} \frac{\partial }{\partial x}\left[\begin{array}{l} \frac{Y}{1-\mu^{2}}\frac{\partial }{\partial x}u_{1}^{k}+\frac{Y}{1-\mu^{2}}\left(ik\cos\theta \right)u_{1}^{k}\\ +\frac{Y\mu }{1-\mu^{2}}\frac{\partial }{\partial y}u_{2}^{k}+\frac{Y\mu }{1-\mu^{2}}\left(ik\sin\theta \right)u_{2}^{k} \end{array}\right]+\frac{\partial }{\partial y}\left[\begin{array}{l} \frac{Y}{2\left(1+\mu \right)}\frac{\partial }{\partial y}u_{1}^{k}+\frac{Y}{2\left(1+\mu \right)}\left(ik\sin\theta \right)u_{1}^{k}\\ \frac{Y}{2\left(1+\mu \right)}\frac{\partial }{\partial x}u_{2}^{k}+\frac{Y}{2\left(1+\mu \right)}\left(ik\cos\theta \right)u_{2}^{k} \end{array}\right]\\ +\frac{Y}{1-\mu^{2}}\left(ik\cos\theta \right)\frac{\partial }{\partial x}u_{1}^{k}+\frac{Y}{2\left(1+\mu \right)}\left(ik\sin\theta \right)\frac{\partial }{\partial y}u_{1}^{k}+\frac{Y}{2\left(1+\mu \right)}\left(ik\sin\theta \right)\frac{\partial }{\partial x}u_{2}^{k}+\frac{Y\mu }{1-\nu^{2}}\left(ik\cos\theta \right)\frac{\partial }{\partial y}u_{2}^{k}\\ +\left[\frac{Y}{1-\mu^{2}}{\left(ik\cos\theta \right)}^{2}+\frac{Y}{2\left(1+\mu \right)}{\left(ik\sin\theta \right)}^{2}\right]u_{1}^{k}+\left[\frac{Y\mu }{1-\mu^{2}}+\frac{Y}{2\left(1+\mu \right)}\right]\left(ik\sin\theta \cdot ik\cos\theta \right)u_{2}^{k}+\left(-\rho \omega^{2}\right)u_{1}^{k}=0 \end{array}\\ \begin{array}{l} \frac{\partial }{\partial x}\left[\begin{array}{l} \frac{Y}{2\left(1+\mu \right)}\frac{\partial }{\partial y}u_{1}^{k}+\frac{Y}{2\left(1+\mu \right)}\left(ik\sin\theta \right)u_{1}^{k}\\ +\frac{Y}{2\left(1+\mu \right)}\frac{\partial }{\partial x}u_{2}^{k}+\frac{Y}{2\left(1+\mu \right)}\left(ik\cos\theta \right)u_{2}^{k} \end{array}\right]+\frac{\partial }{\partial y}\left[\begin{array}{l} \frac{Y\mu }{1-\mu^{2}}\frac{\partial }{\partial x}u_{1}^{k}+\frac{Y\mu }{1-\mu^{2}}\left(ik\cos\theta \right)u_{1}^{k}\\ +\frac{Y}{1-\mu^{2}}\frac{\partial }{\partial y}u_{2}^{k}+\frac{Y}{1-\mu^{2}}\left(ik\sin\theta \right)u_{2}^{k} \end{array}\right]\\ +\frac{Y\mu }{1-\mu^{2}}\left(ik\sin\theta \right)\frac{\partial }{\partial x}u_{1}^{k}+\frac{Y}{2\left(1+\mu \right)}\left(ik\cos\theta \right)\frac{\partial }{\partial y}u_{1}^{k}+\frac{Y}{2\left(1+\mu \right)}\left(ik\cos\theta \right)\frac{\partial }{\partial x}u_{2}^{k}+\frac{Y}{1-\mu^{2}}\frac{\partial }{\partial y}\left(ik\sin\theta \right)u_{2}^{k}\\ +\left[\frac{Y}{2\left(1+\mu \right)}+\frac{Y\mu }{1-\nu^{2}}\right]\left(ik\sin\theta \cdot ik\cos\theta \right)u_{1}^{k}+\left[\frac{Y}{2\left(1+\mu \right)}{\left(ik\cos\theta \right)}^{2}+\frac{Y}{1-\mu^{2}}{\left(ik\sin\theta \right)}^{2}\right]u_{2}^{k}+\left(-\rho \omega^{2}\right)u_{2}^{k}=0 \end{array} \end{array}$$

## Appendix B. Parameter Substitution Conversion Form (Piezo-Electric Sheet)

$$\begin{array}{c} \begin{array}{l} \frac{\partial }{\partial x}\left[\begin{array}{l} \frac{s_{11}^{E^{\prime }}}{{\left(s_{11}^{E^{\prime }}\right)}^{2}-{\left(s_{11}^{E^{\prime }}\right)}^{2}}\frac{\partial }{\partial x}u_{1}^{k}+\frac{s_{11}^{E^{\prime }}}{{\left(s_{11}^{E^{\prime }}\right)}^{2}-{\left(s_{11}^{E^{\prime }}\right)}^{2}}\left(ik\cos\theta \right)u_{1}^{k}\\ +\frac{-s_{12}^{E^{\prime }}}{{\left(s_{11}^{E^{\prime }}\right)}^{2}-{\left(s_{11}^{E^{\prime }}\right)}^{2}}\frac{\partial }{\partial y}u_{2}^{k}+\frac{-s_{12}^{E^{\prime }}}{{\left(s_{11}^{E^{\prime }}\right)}^{2}-{\left(s_{11}^{E^{\prime }}\right)}^{2}}\left(ik\sin\theta \right)u_{2}^{k} \end{array}\right]+\frac{\partial }{\partial y}\left[\begin{array}{l} \frac{1}{s_{66}^{E}}\frac{\partial }{\partial y}u_{1}^{k}+\frac{1}{s_{66}^{E}}\left(ik\sin\theta \right)u_{1}^{k}\\ \frac{1}{s_{66}^{E}}\frac{\partial }{\partial x}u_{2}^{k}+\frac{1}{s_{66}^{E}}\left(ik\cos\theta \right)u_{2}^{k} \end{array}\right]\\ +\frac{s_{11}^{E^{\prime }}}{{\left(s_{11}^{E^{\prime }}\right)}^{2}-{\left(s_{11}^{E^{\prime }}\right)}^{2}}\left(ik\cos\theta \right)\frac{\partial }{\partial x}u_{1}^{k}+\frac{1}{s_{66}^{E}}\left(ik\sin\theta \right)\frac{\partial }{\partial y}u_{1}^{k}+\frac{1}{s_{66}^{E}}\left(ik\sin\theta \right)\frac{\partial }{\partial x}u_{2}^{k}+\frac{-s_{12}^{E^{\prime }}}{{\left(s_{11}^{E^{\prime }}\right)}^{2}-{\left(s_{11}^{E^{\prime }}\right)}^{2}}\left(ik\cos\theta \right)\frac{\partial }{\partial y}u_{2}^{k}\\ +\left[\frac{s_{11}^{E^{\prime }}}{{\left(s_{11}^{E^{\prime }}\right)}^{2}-{\left(s_{11}^{E^{\prime }}\right)}^{2}}{\left(ik\cos\theta \right)}^{2}+\frac{1}{s_{66}^{E}}{\left(ik\sin\theta \right)}^{2}\right]u_{1}^{k}+\left[\frac{-s_{12}^{E^{\prime }}}{{\left(s_{11}^{E^{\prime }}\right)}^{2}-{\left(s_{11}^{E^{\prime }}\right)}^{2}}+\frac{1}{s_{66}^{E}}\right]\left(ik\sin\theta \cdot ik\cos\theta \right)u_{2}^{k}+\left(-\rho \omega^{2}\right)u_{1}^{k}=0 \end{array}\\ \begin{array}{l} \frac{\partial }{\partial x}\left[\begin{array}{l} \frac{1}{s_{66}^{E}}\frac{\partial }{\partial y}u_{1}^{k}+\frac{1}{s_{66}^{E}}\left(ik\sin\theta \right)u_{1}^{k}\\ +\frac{1}{s_{66}^{E}}\frac{\partial }{\partial x}u_{2}^{k}+\frac{1}{s_{66}^{E}}\left(ik\cos\theta \right)u_{2}^{k} \end{array}\right]+\frac{\partial }{\partial y}\left[\begin{array}{l} \frac{-s_{12}^{E^{\prime }}}{{\left(s_{11}^{E^{\prime }}\right)}^{2}-{\left(s_{11}^{E^{\prime }}\right)}^{2}}\frac{\partial }{\partial x}u_{1}^{k}+\frac{-s_{12}^{E^{\prime }}}{{\left(s_{11}^{E^{\prime }}\right)}^{2}-{\left(s_{11}^{E^{\prime }}\right)}^{2}}\left(ik\cos\theta \right)u_{1}^{k}\\ +\frac{s_{11}^{E^{\prime }}}{{\left(s_{11}^{E^{\prime }}\right)}^{2}-{\left(s_{11}^{E^{\prime }}\right)}^{2}}\frac{\partial }{\partial y}u_{2}^{k}+\frac{s_{11}^{E^{\prime }}}{{\left(s_{11}^{E^{\prime }}\right)}^{2}-{\left(s_{11}^{E^{\prime }}\right)}^{2}}\left(ik\sin\theta \right)u_{2}^{k} \end{array}\right]\\ +\frac{-s_{12}^{E^{\prime }}}{{\left(s_{11}^{E^{\prime }}\right)}^{2}-{\left(s_{11}^{E^{\prime }}\right)}^{2}}\left(ik\sin\theta \right)\frac{\partial }{\partial x}u_{1}^{k}+\frac{1}{s_{66}^{E}}\left(ik\cos\theta \right)\frac{\partial }{\partial y}u_{1}^{k}+\frac{1}{s_{66}^{E}}\left(ik\cos\theta \right)\frac{\partial }{\partial x}u_{2}^{k}+\frac{s_{11}^{E^{\prime }}}{{\left(s_{11}^{E^{\prime }}\right)}^{2}-{\left(s_{11}^{E^{\prime }}\right)}^{2}}\frac{\partial }{\partial y}\left(ik\sin\theta \right)u_{2}^{k}\\ +\left[\frac{1}{s_{66}^{E}}+\frac{-s_{12}^{E^{\prime }}}{{\left(s_{11}^{E^{\prime }}\right)}^{2}-{\left(s_{11}^{E^{\prime }}\right)}^{2}}\right]\left(ik\sin\theta \cdot ik\cos\theta \right)u_{1}^{k}+\left[\frac{1}{s_{66}^{E}}{\left(ik\cos\theta \right)}^{2}+\frac{s_{11}^{E^{\prime }}}{{\left(s_{11}^{E^{\prime }}\right)}^{2}-{\left(s_{11}^{E^{\prime }}\right)}^{2}}{\left(ik\sin\theta \right)}^{2}\right]u_{2}^{k}+\left(-\rho \omega^{2}\right)u_{2}^{k}=0 \end{array} \end{array}$$
